# Richmond Academy of Medicine and Surgery

**Published:** 1894-04

**Authors:** J. F. Woodward

**Affiliations:** Reporter


### RICHMOND ACADEMY OF MEDICINE AND SURGERY.

MARCH 13, 1894.

*Dr. I. S. Welford, President, in the chair.*

DR. W. T. OPPENHEIMER concluded his paper on "Pressure of Intestinal Gases." He devoted most of his speech to the treatment in these cases, as he had discussed very fully the *causes* and *effects* in a previous paper. For immediate relief he relied more especially upon effervescent alkalies, carbolic acid, creosote and massage. Some one of these remedies generally gave relief, but the real aim of treatment was directed to a means of preventing the formation of these irritating gases by correcting faulty digestion.

DR. W. W. PARKER thought that a more general study of the chemistry of foods would open up new modes of combating this trouble.

DR. I. S. WELFORD spoke of the utility of salol, five grains, charcoal five grains, just after meals. He did not believe in the careless use of digestive ferments. That pepsin only acted successfully in presence of an acid, and that pancreatine was useless if given while stomachic digestion was in progress.

DR. L. B. EDWARDS spoke of the relief so frequently obtained by the use of the intestinal and stomach tubes.

DR. JAS. N. ELLIS recognized the efficiency of the measures suggested by Dr. Edwards for the removal of gases from the gastro-intestinal tract, but realized that objections would be offered by the patient to the introduction of stomach or rectal tubes, and thought some less repulsive means worthy of consideration. He thought the object of our treatment should be first to relieve existing distension, and second to prevent subsequent accumulations. That lime water is a very simple and efficient agent for immediate removal of one of the most common causes of gastro-intestinal distension, its action depending upon the well-known affinity of $CO_2$ for calcium. The chemical reaction (which results in the formation of two harmless compounds, carbonate of lime and water) may be indicated as follows: $CO_2 + Ca\,2HO = Ca\,CO_3 + H_2O$. A similar reaction occurs when the gas happens to be $H_2S$, sulphide of calcium and $H_2O$ resulting. For the prevention of subsequent accumulation of these and other gases consequent upon fermentative dyspepsia, the means are equally simple and effective when confined to stomach or upper part of small intestine; an emulsion containing one grain of carbolic acid to the dose, taken at meal time, will be found useful. When involving the large or adjacent part of small intestine, *salol* is preferable, as here it is broken up into its different elements. This combined, as Dr. Welford suggested, with charcoal, acted very promptly.

Under report of cases, Dr. Richard Cunningham reported a case he had operated on for convulsions, following a blow on the head.

Patient, negro man, age thirty, miner, fourteen months ago struck on left side of head with a fire-brick; was senseless for nearly four days, then recovered consciousness, and had numerous fits; six days after blow was operated upon for depressed fracture of skull. Previous to operation moderate weakness of right side, fits confined to that side, was aphasic, but recovered speech in one week after operation; fits also lessened, then occurring about twice a week.

*Character of Fit.*—First slight pain in stomach, right thumb begins to work (*i. e.* is approximated to index and middle fingers), then fingers to work, followed by alternate contraction and relaxation of fore-arm, arm and neck, followed by loss of speech; no loss of consciousness, but *motor aphasia.* Fit rarely spreads to left side, and even then does not lose consciousness.

*Physical Examination.*—Negative except following: Right naso-labial fossa slightly flattened. Right little and ring fingers kept moderately extended (ulnar). Right grasp weaker—right 85, left 110. No ocular symptoms; fundus normal. Muscular co-ordination good. Could play banjo well.

*Operation.*—Head shaved. Antiseptic dressings applied day before the operation. Day of operation head reshaved and washed in ether, followed by bichloride 1–2000. Incision to the bone, first one and a half inches, above front eminence, backwards to two inches extent to occiput in a curved line. Trephine (one and a fourth inches) applied one-fourth inch anterior to line of fissure, of Rolando, and about genu of same opening. The hole was enlarged with the Ranguer, also all of old roughened bone at site of fracture and previous operation removed. Hemorrhage checked with prepared wax; *dura mater* opened; no apparent change in cortex, though several adhesions between *dura* and *pia*. These were broken with dural separator, which was always passed under the dura, over the whole motor area. The dura was sutured with catgut. Wound in skin sutured with continuous suture and aseptic dressing applied. Day after operation two slight fits; temperature 38° C., pulse 90. Four weeks later one slight fit, but none up to present time.

DR. GEO. BEN JOHNSON reported a successful operation for omental hernia. Patient, age fifty-one, when first seen had a severe cough, so that the doctor thought it unsafe to operate. Later he operated (he described it as a large pyriform mass extending through inguinal canal down into the scrotum, *irreducible omental hernia*). After thorough antiseptic precautions, he cut down over the hernial sac, finding sac thickened and indurated, containing a large mass of omentum folded upon itself which, when the sac was opened, unfolded like a fan. There were some very large vessels in the neck and a few adhesions, which were either detached with fingers or ligated off. He ligated the neck in sections, including one or more vessels in each section. The mass was separated and the stump replaced in the abdomen. Bassini's modification was then done, transplanting cord and making new sheath for it, wound closed with four stitches of kangaroo silk, and spika bandage adjusted. No food was given for forty-eight hours, then some buttermilk and broth were given. He did not give, contrary to his custom, a laxative for five days. At this time he managed to get satisfactory motions with salts and enemata. On following day temperature 100° F.; ten days later wound entirely healed, when he applied adhesive straps and in a few days, as a matter of precaution put on a soft truss, which was worn only a few days. So far the result has been good; a perfect cure is expected. The doctor exhibited the large mass of omentum removed which had undergone lipomatous or fibro-lipomatous degeneration.

J. F. WOODWARD, Reporter.

---

### IODINE IN TYPHOID FEVER.

Considerable success has attended the treatment of patients suffering from typhoid fever with iodine, in the practice of Dr. Klietsch, who reports, in the *Munchener Medicinische Wochenschrift*, that out of eighty-one cases treated in this manner only two died. The prescription was: Iodide of potass., from ninety to one hundred and twenty grains; distilled water, peppermint water, *aa*, two and one-half drachms; solution of iodine, from eight to twelve grains; from eight to ten drops to be taken every two hours. From four to six days after this treatment was commenced the temperature began to sink, and grave cases became transformed into mild ones. As a rule, defervescence occurred between the eighth and twelfth days; the condition of the bowels was very much better than under other forms of treatment. It was noticed that the appearance of the typhoid rash was exceptional. The theory of the method is that iodate of sodium is formed, and that this is decomposed in the Peyer's patches, giving off nascent iodine, which is inimical to the development of the typhoid bacilli.—*Lancet.*

